# Analysis of HIV pre-exposure prophylaxis (PrEP) needs and PrEP use in Germany among men who have sex with men

**DOI:** 10.1007/s10389-022-01699-y

**Published:** 2022-03-04

**Authors:** Ulrich Marcus, Daniel Schmidt, Susanne B. Schink, Uwe Koppe

**Affiliations:** Department for Infectious Diseases Epidemiology, Robert Koch-Institute, Berlin, Germany

**Keywords:** HIV pre-exposure prophylaxis, PrEP, Men who have sex with men, Germany

## Abstract

**Aims:**

We aim to estimate the number of HIV pre-exposure prophylaxis (PrEP) users among men having sex with men (MSM) in Germany from 2017 through 2020, and analyse if PrEP needs expressed in 2017 might have been met by the expansion of PrEP until 2020.

**Subject and methods:**

We estimated the number of PrEP users by using drug prescription data and information on on-demand/intermittent PrEP use from online surveys to determine the proportion of on-demand/intermittent and daily PrEP use as well as the average number of PrEP pills used.

The number of MSM in need of PrEP in 2017 was estimated based on four groups defined among respondents to a large online survey, combining respondents with PrEP use intention and respondents indicating substantial sexual risks. The size of each group was estimated based on self-selection biases. MSM with PrEP need in 2017 were compared with the estimated number of persons taking PrEP by June 2020.

**Results:**

We estimated a total of 15,600 to 21,600 PrEP users in Germany by the end of June 2020, corresponding to 40–55% of men with PrEP use intention in 2017. A correlation between the regional distribution of PrEP use intention in 11/2017 and actual PrEP use by 06/2020 suggested an unequal regional distribution of unsatisfied needs. The number of men with unmet PrEP needs ranged between 27,500 and 93,000 in 06/2020.

**Conclusion:**

PrEP use in Germany has increased considerably between 10/2017 and 06/2020, but large regional inequalities persist. PrEP is not yet readily accessible, and there is a need to expand services and encourage uptake.

**Supplementary Information:**

The online version contains supplementary material available at 10.1007/s10389-022-01699-y.

## Introduction

HIV pre-exposure prophylaxis (PrEP) use started officially in Germany in 2016 when the European Medicines Agency (EMA) approved the use of tenofovir–emtricitabine for PrEP (European Medicines Agency 2016). With official approval of this two-drug combination for HIV PrEP, any physician in Germany could prescribe these drugs for PrEP use. Yet accessibility remained severely restricted due to prohibitive costs (approximately € 800/month) in the absence of affordable generics in Germany and no legal alternatives to obtain and import cheaper generics from outside the European Union (Federal Law Gazette (BGBl) 2019).

PrEP activists had discovered and promoted a loophole for accessing affordable generics from abroad that relied mainly on a regulation allowing to import generics from outside of the European Union (EU) to the United Kingdom (UK) for personal use. This allowed importing the drugs into the UK and forwarding them by mail to Germany without customs control. However, the number of people in Germany who used this channel or used other informal sources to obtain drugs for use as PrEP remained limited. In October 2017 and only after the patent for tenofovir–emtricitabine had expired, a pharmacist from Cologne exploited a rarely used clause of the German regulatory framework and provided a cheap generic version of tenofovir–emtricitabine as monthly supply packaged for individual clients for approximately €50 per month, the so-called “blister pack PrEP”. The blister pack PrEP became available through a network of HIV-specialized pharmacies (Koelsche Blister GmbH 2017). The price for this generic version was cut to less than one tenth of the usual pharmacy price of the originator and the already approved generics in Germany. Shortly after the launch of this blister pack PrEP, another pharmaceutical company cut the price of their generic version to about €70 per month, later followed by other generics, which then became available across pharmacies in Germany on private prescription (Apotheke adhoc 2017).

PrEP drugs and laboratory tests associated with PrEP initiation and follow-up were not covered by statutory health insurance up to this point. A new law that contained a right to access PrEP including counselling and testing covered by statutory health insurance (BMG 2019; Bundesgesetzblatt 2019) came into effect in September 2019. Coverage within the framework of the statutory health insurance system is now granted provided that PrEP is prescribed by a physician licensed to prescribe PrEP. This license requires continuing medical education and documented case management of people living with HIV and/or PrEP provision, and is thus feasible mainly for HIV specialists (KBV-GKV Bewertungsausschuss 2019). By 2020, all statutory health insurance companies in Germany covered the costs of PrEP when prescribed by licensed PrEP prescribers.

The primary aim of this analysis is to estimate overall PrEP need of MSM in Germany, and how much of this need had been met by 2020. Since no centralized source collects data on PrEP use in Germany, our first step draws upon different sources to estimate (1) the number of PrEP users and (2) the type of PrEP use in Germany between 2017 and 2020. Combining this information, we analyse the extent of needs expressed in 2017 that might have been met by expanding access to PrEP until 2020. Lastly, we investigate whether the number of physicians licensed to prescribe PrEP under the statutory health insurance scheme might still represent a barrier to accessing PrEP after financial barriers have been removed through coverage of drugs and tests by statutory health insurance. Since all available information suggests a marginal use of PrEP by people other than men having sex with men (MSM),^1^ our estimates are restricted to PrEP use among MSM.

## Methods

### Study design and outcomes

#### Estimate the number of PrEP users in Germany between 2017 and 2020. Data sources for the estimate of PrEP users

- Drug prescription data

There is no tracking system for private prescriptions in Germany with the exception of controlled substances. For the time period between October 2017 and August 2019, we obtained data on blister pack PrEP prescriptions at specified time points from the company that repackaged and distributed blister pack PrEP (Koelsche Blister GmbH). These time points were synchronized with the dates when online surveys provided additional information on PrEP access and mode of PrEP use among participating PrEP users.

For the time period starting with September 2019, data on antiretroviral drugs prescribed on statutory health insurance included drugs prescribed for PrEP. We have been using antiretroviral prescription data provided by “Insight Health” (company providing drug prescription data from pharmacies) to determine the number of people treated for HIV in Germany. The method to determine the number of people receiving antiretroviral treatment based on drug prescription data is described in detail elsewhere (Schmidt et al. 2015). Tenofovir disoproxil and emtricitabine (TDF/FTC) is used for PrEP and in HIV treatment alike without the possibility to distinguish its use in the prescription data. To determine the number of defined daily doses (DDD) used for PrEP, we set as baseline the number of DDD prescribed for HIV treatment during the first two quarters of 2019 and designated excess prescriptions as PrEP use from the third quarter 2019 onwards, when PrEP could be prescribed under statutory health insurance rules.

b) Online surveys addressing PrEP use among MSM

The European MSM Internet Survey 2017 (EMIS-2017) addressed PrEP use in all participating European countries. Methods for this low-threshold online survey have been described in detail elsewhere (Weatherburn et al. 2019). In Germany, data for this online survey were mainly collected between early November 2017 and mid-January 2018, with most data collected during early November.

An online survey to specifically address PrEP use among MSM in Germany was designed by the Robert Koch-Institute in 2018 (PrApp-Survey). The survey was rolled out in several rounds: the first round collected data from July to September 2018, the second round from April to mid-June 2019, and the third round from March to early May 2020. Methods for this survey have been described elsewhere (Koppe et al. 2019). In this survey, data on place of residence (not collected in round 1), mode of PrEP access, type of PrEP use (daily vs on-demand) and source of PrEP (informal sources, blister pack PrEP, other self-paid generic, health insurance-covered PrEP) were collected.

iii) Online profiles indicating PrEP use

PlanetRomeo is a globally operating gay dating app and website which is highly popular among MSM in Germany (https://www.planetromeo.com/). According to PlanetRomeo, there were 643,000 active user profiles in Germany by June 2020. Upon request in June 2020, PlanetRomeo provided the number and regional distribution (by federal state) of user profiles from Germany in which “PrEP use” or “PrEP and condom use” were stated as “safer sex” choices.

#### Statistical methods to estimate the number of PrEP users

To estimate the absolute number of PrEP users, we took the data on prescribed monthly doses for daily PrEP use and multiplied this with a factor that results in the number of monthly PrEP users, based on assumptions about 1) the proportions of daily and on-demand/intermittent PrEP use, and 2) the number of on-demand/intermittent PrEP users based on a monthly supply of a daily PrEP dose. Information on 1) and 2) was collected in the online surveys. However, we did not obtain data on how long and often on demand/intermittent PrEP users were taking PrEP. Thus, 2) could not be defined based on data and we had to use plausible assumptions for the calculation of the average number of PrEP pills per month used by intermittent and on-demand users.

#### Estimating the number of MSM in need for PrEP in 2017

In the EMIS-2017 questionnaire respondents were asked “If PrEP was available and affordable to you, how likely would you be to use it?” with five response options ranging from ‘very unlikely’ to ‘very likely’. To assess *subjective* PrEP needs among MSM in Germany we calculated the proportion of survey participants who indicated their willingness to use PrEP (‘quite likely’ or ‘very likely’ = *intention to use PrEP*). To estimate *objective* PrEP needs, we determined the proportion of online survey participants without HIV diagnosis from Germany in EMIS-2017 who reported at least two non-steady partners with whom condomless anal intercourse (CAI) occurred in the previous 12 months.

We analysed the overlap between the *subjective* PrEP needs and the *objective* PrEP needs groups. We defined

- “intention to use PrEP” as having declared an interest to use PrEP;
- “no intention to use PrEP” as not having declared an interest in using PrEP;
- “low sexual risk” as having had less than two non-steady CAI partners within the last 12 months;
- “moderate/high sexual risk” as having had two or more non-steady CAI partners within the last 12 months.

We formed the following four groups:

1. intention to use PrEP, low sexual risk;

Men with intention to use PrEP and less than two non-steady sex partners in the last 12 months were excluded from this group, assuming that most of them indicate a hypothetical need for PrEP in case they would have more partners, or a perceived need based on characteristics of their steady/regular partner;

2) intention to use PrEP, moderate/high sexual risk;
3) no intention to use PrEP, moderate/high sexual risk;
4) no intention to use PrEP, low sexual risk.

Groups 1–3 were defined as in need of/benefitting from PrEP use.

##### Statistical methods

We calculated the proportions of EMIS-2017 respondents that belong to groups 1–3 as described above. To obtain absolute numbers of people in need of PrEP we extrapolated the results to the gay population in Germany. We assumed that 1.5% of the adult male population are gay (Mercer et al. 2016; Haversath et al. 2017). The distribution of the gay population across federal states in Germany was estimated based on the relative federal state distribution of EMIS-2017 respondents (Marcus et al. 2009). Next, we calculated self-selection biases of PrEP users participating in EMIS-2017 and in the three PrApp-Survey rounds. Then we estimated possible ranges for self-selection biases of EMIS-2017 participants for the four groups. We used rounded absolute numbers for the four predefined groups and four different assumptions about self-selection biases: a) a minimal self-selection bias (the relative proportion of the four groups in the gay population is the same as in EMIS-2017), b) and c) two variants of intermediate self-selection biases, based on different weightings for partner numbers and intended PrEP use as main components for determining self-selection bias (we chose weights of a magnitude of 2–2.5 as a combined effect of higher partner numbers and PrEP use intention, which is slightly lower than the empirically derived weight of 3 for EMIS-2017 respondents with a syphilis diagnosis in the previous 12 months), and d) a maximal self-selection bias for men with intended PrEP use, similar to the self-selection bias of actual PrEP users.

#### Analysis how the PrEP needs expressed in 2017 might have been met by expansion of PrEP use until 2020

We compared our estimate of MSM with PrEP needs in 2017 with the estimated number of people taking PrEP by June 2020, assuming that current PrEP users were recruited exclusively from men who indicated PrEP needs in 2017. The group with “PrEP needs met” and the number and proportion of men in need of/potentially benefitting from PrEP who had *no* intention to use PrEP but sexual risks in 2017 were stratified by federal state.

#### Assessing the number of physicians licensed to prescribe PrEP on statutory health insurance in Germany as potential barrier for PrEP uptake

There is no publicly accessible data source for the number of physicians licensed to prescribe HIV drugs for PrEP covered by statutory health insurance in Germany. As a proxy, we searched the website of the German association of physicians in private practice providing HIV-care “Deutsche Arbeitsgemeinschaft niedergelassener Ärzte in der Versorgung HIV-Infizierter e. V. (dagnä) “for the number of HIV specialized care providers by federal state (DAGNÄ 2020). We constructed a “PrEP prescriber density” by calculating the number of potential PrEP prescribers per 10,000 MSM for each federal state. We correlated this density factor with the extent of met PrEP needs.

## Results

### Number of PrEP users in Germany

#### Drug prescription data

By end of October 2017, the company producing blister pack PrEP had filled prescriptions for 513 monthly PrEP doses. In July 2018 this number had increased to 3125 doses, and in May 2019 to 4022 doses [personal communication by E. Tenberken].

By the end of 2019, the number of drug prescriptions assumed to be used for PrEP and covered by statutory health insurance companies had increased to 10,176 monthly doses. For the first two quarters of 2020 we calculated an average of 10,788 monthly prescriptions (see Table 1).

**Table 1:** Data on PrEP access from online surveys in Germany, 2017–2020, and estimation of absolute number of PrEP users

	EMIS-2017	PrApp round 1 (07/2018)	PrApp round 2 (05/2019)	PrApp round 3^1^, PrEP prescription data 12/2019	PrApp round 3^1^, PrEP prescription data 06/2020	Scenario 2** 06/2020 PrEP prescription data 2020, presumed self-pay 20%, informal sources 5%
a) Survey data	%	%	%	%	%	%
Medical prescription in Germany	56	81	83	94	94	95
Blister pack PrEP	56	54	58	n.a.	n.a.	n.a.
Other sources (informal, self-pay non-blister-PrEP generics, trials)	44	46	42	n.a.	n.a.	n.a.
Prescription on statutory health insurance	n.a.	n.a.	5	80	80	*75*
Prescription self-pay (blister pack PrEP + other generics)	56	81	82	14	14	*20*
Informal	44	19	13	6	6	*5*
Proportion PrEP on demand	29	29	30	19	19	*40*
Proportion of PrEP users participating in survey	37%	28%	34%		6%	n.a.
	Scenario 1 (based on survey result)*					Scenario 2 **
b) estimation of absolute number of PrEP users	*N*	*N*	*N*	*N*	*N*	*N*
Number of blister pack PrEP/ private self-pay prescriptions	513	3125	4022	1796	1904	2877
Number of monthly health insurance prescriptions				10,176	10788	10788
Number of PrEP users on prescribed PrEP	660	4018	5171	13,814	14,644	20,497
Number of PrEP users on informal sources/other (non blister pack) self-pay generics	518	3423	3745	882	935	1079
Estimated total number of PrEP users	1178	7440	8916	14,695	15,579	21,576

n.a. = not applicable.

^1^data from PrApp-Survey round 3 was collected in 03/2020

*assumptions scenario 1: 33% (20%) on-demand/intermittent users, three on-demand/intermittent users supported by one full monthly dose

**assumptions scenario 2: 40% on-demand/intermittent users, six on-demand/intermittent users supported by one full monthly dose

#### Survey data

The number of EMIS-2017 respondents living in Germany who indicated current PrEP use was 452, representing 2.1% of the survey respondents, 17 of whom did not provide information on their place of residence. In rounds 1 (July-September 2018), 2 (April/May 2019) and 3 (March-May 2020) of the PrApp-Survey a total of 2118, 3071 and 964 PrEP users participated, of whom 2252 and 790 in rounds 2 and 3 provided information on their place of residence, round 1 did not collect this information (see Table 1).

#### PlanetRomeo PrEP profiles

In June 2020, PlanetRomeo provided information on the geographical distribution of 15,633 user profiles in Germany who had indicated PrEP use (*n* = 9207) or the combination of PrEP and condom use (*n* = 6426) as their “safer sex” preference [personal communication by PlanetRomeo Chief Operating Officer].

For the interpretation of these data we considered two important pieces of information from other sources: 1) the PrApp-Survey provided information that a proportion of 30% PrEP users do not provide PrEP use information in their online profiles. This would argue for an underestimate of PrEP users by the number of PrEP profiles on PlanetRomeo; 2) A survey conducted in 2020 among people diagnosed with HIV and living in Germany found that a substantial proportion (11.5%) of MSM using online dating reported communicating PrEP use to their potential partners^2^ [personal communication by Franziska Hartung]. While the representativeness of these data for MSM diagnosed with HIV is unknown, a proportion of 11.5% of MSM diagnosed with HIV could amount to approximately 3450 PrEP profiles (for the calculation see Table S1). Considering these two biases for the PlanetRomeo profiles, the PrEP profile number on PlanetRomeo would support a number of approximately 17,400 current PrEP users among MSM in Germany.

#### Estimating the number of PrEP users

We defined two scenarios to describe a possible range for the total number of PrEP users in Germany.

In *scenario 1* we make the following assumptions: the proportion of on-demand/intermittent PrEP users was 33% for the time period before PrEP became reimbursable by statutory health insurance and dropped to 20% after September 2019 (based on respective responses in the PrApp-Survey rounds 1–3). Intermittent/on-demand PrEP users will only obtain another prescription when they run out of medication, thus they will be underrepresented in the monthly prescription data. We assumed that the actual number of intermittent/on-demand PrEP users will be on average three-fold higher than the monthly prescriptions for men from these two groups (i.e., a monthly PrEP dose suffices for 3 months intermittent/on-demand PrEP use on average).

In *scenario 2* we assume that: (1) a monthly PrEP dose meets the needs of six on-demand/intermittent PrEP users for 1 month and (2) that — due to a possible systematic survey participation bias — the proportion of on-demand/intermittent PrEP users is 40%, and thus higher than indicated by respondents of the PrApp-Surveys, and that the proportion of self-payers who are not reimbursed by statutory health insurance might also be slightly higher than among the PrApp-Survey respondents.

Data from the PrApp-Surveys on the number of PrEP pills used per months by daily, intermittent, and on-demand users, and on the proportion of participating PrEP users indicating PrEP use on their online profiles is presented in supplemental Tables S2 and S3.

Table 1 shows our calculation of the estimated number of PrEP users in Germany at five different points in time (early November 2017, end of July 2018, end of May 2019, end of December 2019, and end of June 2020) based on drug prescriptions, proportions of daily and on-demand/intermittent PrEP use, assumptions on how many on-demand/intermittent PrEP users are supported by a monthly dose, and the proportion of additional drug sources as indicated by respondents of online surveys. We arrive at estimates of approximately 15,600 PrEP users in Germany by end of June 2020 for scenario 1, and of approximately 21,600 PrEP users in Germany by end of June 2020 for scenario 2.

#### Regional distribution of PrEP users

**Fig. 1:**
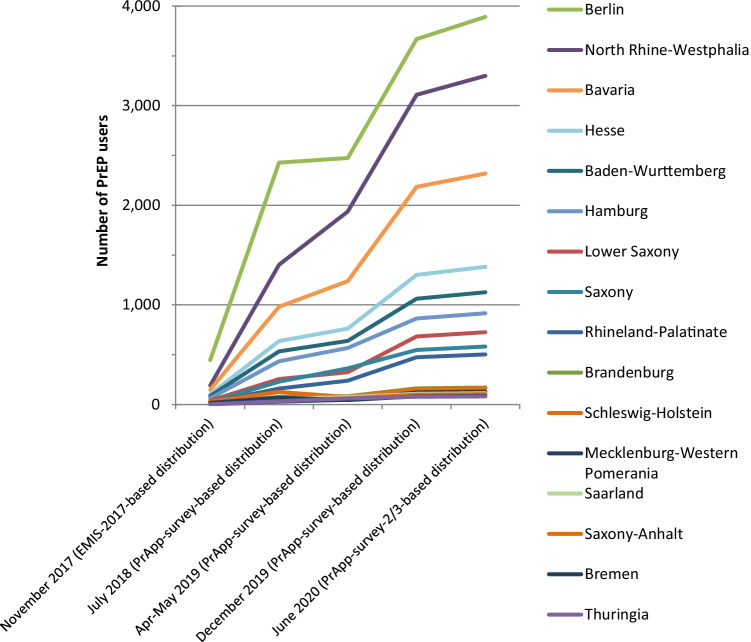
Estimated number of PrEP users at different time points stratified by federal state

Figure 1 and Table 2 provide the estimated absolute numbers of PrEP users by federal state based on the regional distribution in the respective online surveys and the estimated totals from scenario 1 at the time points 1) early November 2017, 2) end of July 2018 (PrApp-Survey round 1), 3) end of April 2019 (PrApp-Survey round 2),4) end of December 2019 (PrApp-Survey round 3 distribution), 5) June 2020 (PrApp-Survey round 2–3 distribution), and for scenario 2 in June 2020 (PrApp-Survey round 2–3 distribution). As a sensitivity analysis, we also provide for comparison of scenario 1 and 2 with a regional distribution based on the PrEP profile distribution of the PlanetRomeo PrEP profiles for June 2020 (PrEP user profile distributions on PlanetRomeo are not available for earlier time points).

**Table 2:** Estimated absolute numbers of PrEP-users by federal state at different time points and for scenario 1 and 2

	Online survey-based distributions						PlanetRomeo profile-based distribution	
	Scenario 1					Scenario 2	Scenario 1	Scenario 2
	November 2017 (EMIS-2017)	July 2018	Apr-May 2019	December 2019	June 2020	June 2020	June 2020	June 2020
Baden-Wurttemberg	86	533	639	1061	1126	1560	990	1371
Bavaria	149	982	1239	2184	2318	3210	2146	2972
Berlin	447	2427	2474	3668	3892	5389	4590	6356
Brandenburg	14	78	84	162	172	238	155	214
Bremen	17	70	44	82	87	121	148	204
Hamburg	63	435	568	864	917	1269	952	1318
Hesse	102	637	763	1302	1382	1914	1198	1659
Mecklenburg-Western Pomerania	3	28	48	123	130	180	95	131
Lower Saxony	39	256	324	684	725	1004	678	938
North Rhine-Westphalia	190	1403	1939	3109	3299	4568	3197	4427
Rhineland-Palatinate	19	160	239	475	504	698	456	631
Saarland	8	57	76	108	115	159	140	193
Saxony	25	229	364	548	582	806	431	597
Saxony-Anhalt	6	44	64	98	105	145	123	170
Schleswig-Holstein	30	126	76	155	165	228	211	292
Thuringia	3	34	60	77	82	113	91	126
Total number of PrEP users	1200	7500	9000	14,700	15,600	21,601	15,600	21,601

**Fig. 2:**
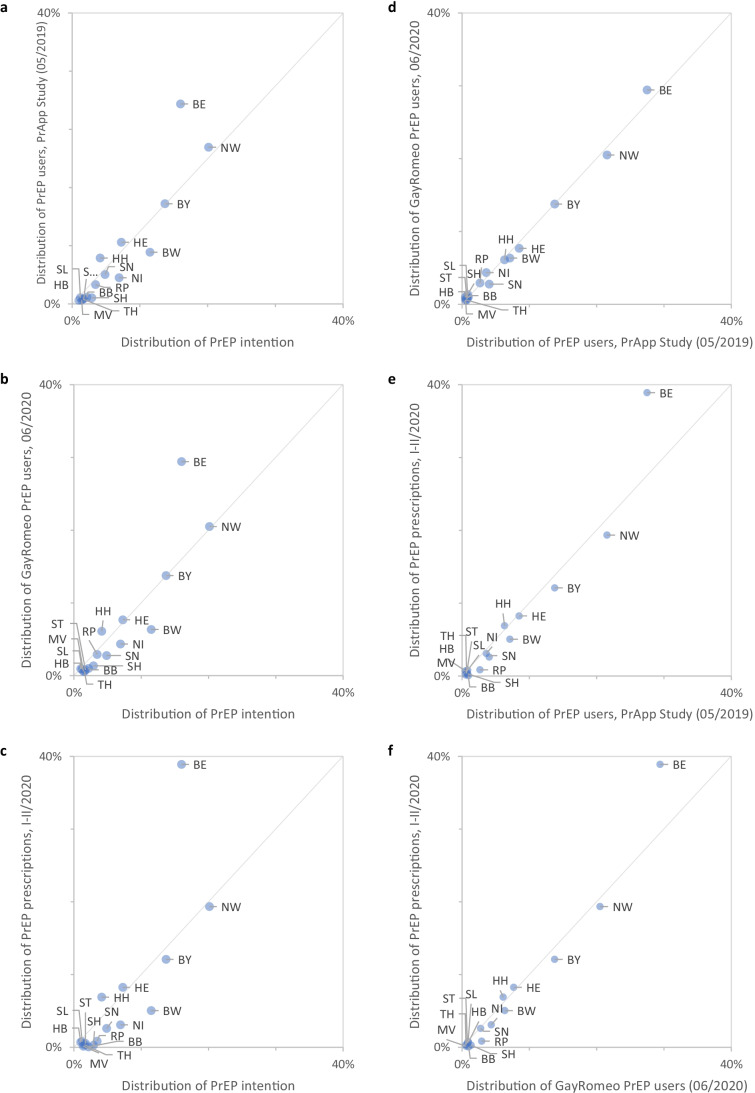
**a–c**) Correlation of the regional distribution of PrEP use intention in 2017 with PrEP use in 2020 as indicated in the PrApp Study, in GayRomeo PrEP profiles, and by statutory health insurance PrEP prescriptions in 2020; **d–f**) Correlation of the regional distribution of PrEP users in 2020 between PrApp survey respondents and GayRomeo PrEP profiles, and between PrApp Study respondents and GayRomeo PrEP profiles with statutory health insurance PrEP prescriptions

Figure 2 shows six correlation graphs, demonstrating high correlations of the proportional distribution by federal state in Germany of EMIS-2017 participants using PrEP, PrApp-Survey participants using PrEP, PlanetRomeo PrEP profiles, and PrEP prescription data. However, the correlations between the state distribution of PrApp-Survey participants using PrEP and PrEP prescription data as of end of 2019 and for 2020 show major deviations for Berlin, with a higher proportion of prescriptions in Berlin (38.9% in the first two quarters of 2020) compared to the proportion of PrApp-Survey participants using PrEP [27.5% in round 2 (05/2019) and 22.4% in round 3 (03/2020)].

Table S4 provides the proportional regional distributions (by federal state) of all EMIS-2017 participants, EMIS-2017 participants using PrEP, PrApp-Survey participants using PrEP, all PlanetRomeo profiles in Germany, and PlanetRomeo PrEP profiles.

### Estimating PrEP needs in Germany

To estimate the total size of the four PrEP needs groups in the MSM population in Germany, we made assumptions about self-selection biases for these groups in EMIS-2017. Based on the PrEP user estimates at different time points, we determined that 30–35% of all PrEP users at these time points in Germany participated in EMIS-2017 and the first two rounds of the PrApp-Survey surveys, suggesting very high self-selection biases for actual PrEP users (see Table 1). For comparison, 20,000 male participants not diagnosed with HIV took part in EMIS-2017, which corresponds to almost 6% of the estimated non-HIV-diagnosed population of gay men living in Germany (*N* = 350,000, 1.5% of adult males aged 15–64).

We assume that the intention to use PrEP is associated with a self-selection bias that lies in-between the self-selection bias for PrEP users (30-35%) and the average self-selection bias for survey participants (6%). We also assume that the size of the bias is associated with factors such as PrEP use intention, partner numbers, self-perceived risk, and PrEP awareness — the key factors we identified in a multivariable regression analysis (see Table S5). We chose weights of a magnitude of 2–2.5 as a combined effect of higher partner numbers and PrEP use intention, which is slightly lower than the empirically derived weight of 3 for EMIS-2017 respondents with a syphilis diagnosis in the previous 12 months. The distribution of partner numbers, PrEP awareness, and recency of HIV testing that we consider a surrogate for self-perceived risk in this context in the four groups is shown in Fig. S1.

Table S6 provides absolute data for the size of the four PrEP need groups in EMIS-2017.

Based on these assumptions we provide range estimates for the sizes of all four PrEP need groups in Table 3. The minimum size for the PrEP need groups would reflect a self-selection bias similar to actual PrEP users, the maximum size would reflect no specific self-selection bias for PrEP use intention. The two intermediate variants represent two different — arbitrary — assumptions of intermediate self-selection biases. The estimated PrEP need ranges between 49,500 and 109,000 men, the unsatisfied PrEP need between 33,500 and 93,000 men for scenario 1 as of June 2020, and between 27,500 and 87,000 for scenario 2, assuming no change in PrEP needs between end of 2017 and June 2020, and using a total population size estimate of 350,000 adult gay men not diagnosed with HIV living in Germany (16).

**Table 3:** Estimating the size range of PrEP need groups in Germany

	Intention, no reported risk*	Intention and risk	No intention, but risk	No intention, no reported risk		Estimated PrEP need	Unmet PrEP need (scenario 1, 16,000 PrEP users)	Unmet PrEP need (scenario 2, 22,000 PrEP users)
Absolute number, assumption: no self-selection bias	40,000	37,000	32,000	241,000		109,000	93,000	87,000
Self-selection	6%	6%	6%	6%				
Absolute number, assumption: medium self-selection bias, determined by PrEP use intention > partner numbers (variant 1)	22,000*	17,000*	27,000**	284,000		66,000	50,000	44,000
Self-selection	11%	13%	7%	5%				
Absolute number, assumption: medium self-selection bias, mainly determined by partner numbers > PrEP use intentions (variant 2)	20,000	15,000	17,000	298,000		52,000	36,000	30,000
Self-selection	12%	15%	11%	5%				
Absolute number, assumption: extreme self-selection, only determined by PrEP use intention	7500	7000	35,000	300,500		49,500	33,500	27,500
Self-selection	31%	32%	5%	5%				

* N with intention in Table 4

**N with risk, no intention in Table 4

### Comparison of expressed PrEP need in 2017 with PrEP use in 2020

In the next step, we compare how this estimated PrEP need from 2017 compares to the number of estimated PrEP users in 2020. To calculate a regional PrEP need distribution in Table 4, we used the totals from variant 1 shown in Table 3 and apply it to the scenario 1 total of current PrEP users (*N* = 15,600) and the scenario 2 total of current PrEP users (*N* = 21,600). The variant 1 estimate assumes that PrEP use intention and the number of sex partners in the last 12 months are the main determinants for the selection bias.

**Table 4:** Regional need distribution und unmet needs by federal states

			Scenario 1*				Scenario 2*			
	*N* with intention (variant 1 from Table 3)	*N* with risk, no intention (variant 1 from Table 3)	PrEP-user =15,600	% of intentional users actually using PrEP	In need of PrEP^	% in need with no intention (demand generation required)	PrEP-user = 21,600	% of intentional users actually using PrEP	In need of PrEP^	% in need with no intention (demand generation required)
Baden-Wurttemberg	4494	2978	1126	25.1%	6346	46.9%	1560	34.7%	5912	50.4%
Bavaria	5382	3276	2318	43.1%	6340	51.7%	3210	59.6%	5448	60.1%
Berlin	6140	4215	3892	63.4%	6462	65.2%	5389	87.8%	4966	84.9%
Brandenburg	857	432	172	20.0%	1117	38.7%	238	27.8%	1051	41.1%
Bremen	392	357	87	22.3%	662	54.0%	121	30.9%	628	56.8%
Hamburg	1614	1355	917	56.8%	2053	66.0%	1269	78.6%	1700	79.7%
Hesse	2846	1966	1382	48.6%	3430	57.3%	1914	67.3%	2898	67.8%
Mecklenburg-Western Pomerania	548	477	130	23.8%	895	53.3%	180	32.8%	845	56.4%
Lower Saxony	2726	1683	725	26.6%	3684	45.7%	1004	36.8%	3405	49.4%
North Rhine-Westphalia	7871	5600	3299	41.9%	10,171	55.1%	4568	58.0%	8903	62.9%
Rhineland-Palatinate	1341	745	504	37.6%	1582	47.1%	698	52.1%	1388	53.7%
Saarland	471	402	115	24.3%	759	53.0%	159	33.8%	714	56.3%
Saxony	1901	1325	582	30.6%	2644	50.1%	806	42.4%	2420	54.8%
Saxony-Anhalt	668	506	105	15.6%	1070	47.3%	145	21.7%	1029	49.2%
Schleswig-Holstein	1132	1087	165	14.5%	2054	52.9%	228	20.1%	1991	54.6%
Thuringia	617	596	82	13.3%	1131	52.7%	113	18.3%	1100	54.2%
Total	39,000	27,000	15,600	40.0%	50,399	53.6%	21,600	55.4%	44,400	60.8%

* regional distribution according to PrEP-user distribution in PrApp-Surveys round 2

^ includes men with intention to use PrEP and/or with reported sexual risk minus those men already using PrEP.

**Fig. 3:**
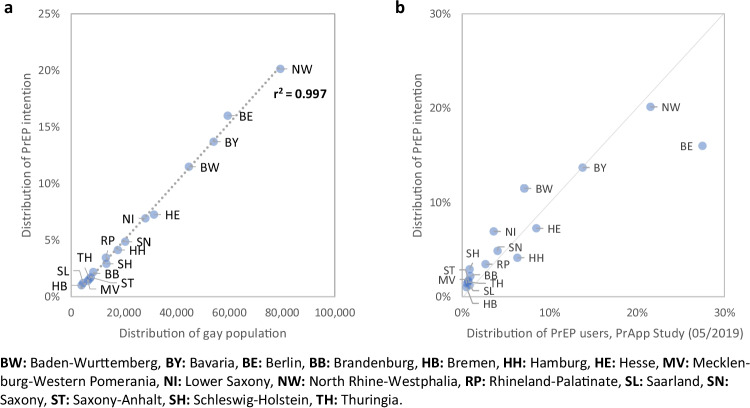
**a**) Correlation between the regional distribution of PrEP use intention in 2017 and the regional distribution of the gay population in Germany; **b**) Correlation between regional distribution of PrEP use intention in 2017 and PrEP use as indicated by the PrApp Survey in 05/2019

Table 4 shows proportions of satisfied intention to use PrEP and unmet needs with no intention to use PrEP by federal state by June 2020. Figure 3a shows the almost perfect correlation between the regional distribution of intention to use PrEP in November 2017 and the distribution of the gay population in Germany, while Fig. 3b shows how intention to use PrEP correlates with actual PrEP use by mid-2020, suggesting an unequal regional distribution of unmet needs, regardless which of the two scenarios is used.

### Analysis of PrEP prescriber density and correlation with PrEP use

**Fig. 4:**
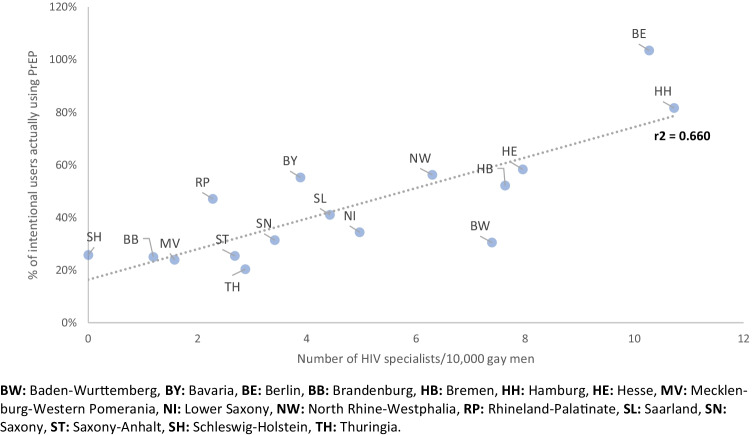
Correlation between the number of PrEP prescribers per 10,000 gay population by federal state and PrEP needs met as estimated using scenario 2 PrEP user estimate and variant 1 PrEP need estimate

There were a total of 246 licensed HIV specialists listed on the DAGNÄ website in early 2021. We use this information as proxy for the number of licensed PrEP prescribers in Germany. Since this number differs regionally by federal state, it might explain the unequal distribution of unsatisfied PrEP needs. Thus, we analysed the correlation between satisfied PrEP needs and number of HIV specialists per 10,000 gay men practicing in the respective federal state, and found a strong correlation between these two parameters (Fig. 4).

## Discussion

We estimated absolute numbers of MSM using PrEP and their regional distribution in Germany between November 2017 and June 2020. Based on drug prescription data and self-reported PrEP use, the number of PrEP users in Germany during this period increased more than tenfold, but over time the increase appears to slow down. After an initial rapid increase in the number of PrEP users when affordable generics became available, we see a short phase of levelling off, and a second phase of rapid increase after PrEP, including associated tests, became reimbursable by statutory health insurance in Germany. The second rapid increase phase suggests that statutory health insurance reimbursement successfully removed an important PrEP access barrier. In 2020 we observed a second levelling-off of the increase, probably explained by behavioural adaptation to the SARS-CoV-2 (Coronavirus) pandemic, and in some areas probably also PrEP demand having been satisfied. Unfortunately, only sparse data on sexual behaviour of German MSM during the SARS-CoV-2 pandemic in 2020 is available. A PlanetRomeo survey from early April 2020 (during the first country-wide lockdown) with approximately 36,000 respondents from Germany showed dramatic reductions of sexual activity with non-steady partners (Planet Romeo 2020). A small survey among PrEP users conducted on Facebook in May 2020 showed reductions of daily PrEP use and increased on-demand use, likely reflecting a reduction of partner numbers (Robert Koch-Institut 2020).

### Validity of assumptions

We focus now on the validity of the assumptions used for the two scenarios to estimate the total number of PrEP users in Germany:

For both scenarios we would like to discuss primarily the validity of the (PrApp-Survey-derived) proportions of on-demand/intermittent use and the validity of the regional distribution, particularly the proportion of PrEP users from Berlin. To estimate proportions of on-demand/intermittent use and regional distribution of PrEP users, we used data on PrEP use from three consecutive online surveys among MSM in Germany. In general, health and sexual behaviour online surveys among MSM such as EMIS-2017 provide reliable data on proportional regional and age distribution (Marcus et al. 2009; Scholz et al. 2019). However, mono-thematic online surveys (such as the PrApp-Surveys) may be affected by more unpredictable participation biases, determined by (for example) unmet needs. Robust evidence for a large online survey participation bias are the very similar proportions of EMIS-2017 respondents and PlanetRomeo user profiles indicating PrEP use in November 2017 and June 2020, despite the more than tenfold increase of PrEP users (see supplemental Table S4). We tried to calibrate online survey data with PrEP use and prescription data from other sources. From these calibrations, we can conclude that self-selection biases for PrEP users to participate in online surveys can be very high and can vary considerably between surveys, depending probably on various factors such as framing of the survey, recruitment methods and advertising of the survey, perceived relevance to individuals, community mobilisation around PrEP access, and competing social and health issues. In our experience, the proportion of estimated PrEP users in Germany participating in online surveys varied between 37% in 2017 and 6% of all MSM actually using PrEP in 2020 (see Table 1). The low participation rate in spring 2020 is probably explained by a reduced intensity of recruitment activities, the coincidence with Coronavirus lockdowns and the overwhelming dominance of the Coronavirus pandemic in public perception and discourse. It is thus conceivable that participation biases could also affect the proportions of on-demand/intermittent PrEP use reported, and differ between regions with high satisfied demand and easy access to PrEP such as Berlin, and regions with higher access barriers. This could result in disproportional lower participation of PrEP users from Berlin in the PrApp-Surveys, as well as disproportional lower participation of on-demand/intermittent PrEP users in the consecutive PrApp-Survey rounds.

The participation bias in Berlin would reduce the discrepancy between the PrApp-Survey-derived PrEP user proportion and the prescription-derived distribution, and would thus be more consistent with the alternative explanations for these discrepancies.

To assess the plausibility of scenario 2, we have to discuss primarily the probability that the total number of PrEP users exceeds the estimate of scenario 1 as suggested by the calculated number of PrEP user profiles on PlanetRomeo. While the number of PlanetRomeo profiles indicating PrEP use is almost identical to the estimate of scenario 1, this coincidence fails to consider that — as outlined above — a proportion of 30% of PrEP users does not include PrEP use information in their online profiles. However, we must consider that a relevant proportion of PrEP profiles might belong to men already diagnosed with HIV who prefer to state in their profile that they take antiretroviral drugs for prevention rather than for treatment. Taking these two pieces of information into account, the PrEP profile number on PlanetRomeo supports that there are approximately 17,400 current PrEP users among MSM in Germany, a number that falls between our scenario 1 and 2 estimates. This number could be even higher if we consider that not all PrEP users may have a profile on PlanetRomeo. Furthermore, abandoned profiles, people creating multiple profiles, and former PrEP users not updating their profile information could lead to an overestimation of PrEP use based on the number of online profiles.

### Regional distribution of PrEP users

Our regional distribution estimates reveal a distinct discrepancy with regard to the number and proportion of PrEP users in Berlin between different data sources, most pronounced between PrApp-Survey data and statutory health insurance data on prescriptions.

In the prescription data, prescriptions are allocated to the geographical area of the prescribing care provider. This results in well-known geographical biases for HIV drug prescription data, since people living in rural and underserved areas usually attend HIV specialists in urban centres for HIV treatment or PrEP prescription. This bias is particularly pronounced in the prescription data for Berlin and Hamburg where the number of prescriptions is thus higher, and is reversed in the surrounding federal states of Schleswig-Holstein, Lower Saxony, Mecklenburg-Western Pomerania, Brandenburg, Saxony-Anhalt, and Thuringia. However, if we assume that the discrepancy between the proportion of PrEP users in the PrApp-Survey and the prescription data is fully explained by this bias, we would have to assume large shifts from other federal states to Berlin to obtain prescriptions there. This is not impossible, but, given its magnitude, appears implausible. We find it more plausible to account for this difference between the PrApp-Survey and prescription data-derived regional distribution for Berlin by a combination of drug prescription shift to Berlin and a disproportional self-selection bias of PrEP users from Berlin in the PrApp-Survey. This could be the case if we assume that the comparatively high levels of satisfied PrEP need reached in Berlin would negatively impact the participation rate in a mono-thematic PrEP-focussed survey.

Thus, we believe it is likely that neither prescription data nor the PrApp-survey data represent the proportion of PrEP users living in Berlin correctly. The proportions observed in the PlanetRomeo PrEP profiles might be closer to the real distribution than PrApp-Survey and drug prescription data, as long as we can safely assume that PrEP profiles of men already diagnosed with HIV do not introduce a specific regional bias. PrEPuser profiles on GayRomeo represent a larger fraction of PrEP users in Germany than PrApp survey samples; the limitations inherent in the distribution of regional prescription data have been discussed. If any, one could assume that reporting PrEP use instead of treatment might be preferred in areas with higher levels of HIV-associated stigma, i.e., outside large cities, thus further increasing — rather than reducing — the regional inequalities that we identified.

Another possible reason for unreliability of regional distribution of drug prescription data is the inability to distinguish between prescriptions for HIV treatment and prescriptions for PrEP use. As described above, we designated excess prescriptions of TDF/FTC from the third quarter 2019 onwards — assuming no relevant changes in the amount of TDF/FTC used for HIV treatment — as PrEP prescriptions. If TDF/FTC use in HIV treatment developed differently in some regions, this would affect the regional distribution.

### Self-selection biases

If our assumptions on self-selection biases are correct, different self-selection biases in countries with different social and political contexts must be considered for multi-country surveys such as EMIS-2017, which would have consequences for comparability of estimates for PrEP use and PrEP needs across countries (Hayes et al. 2019).

It is also difficult to estimate the self-selection biases for survey participants intending to use PrEP or “in need of PrEP” based on number of reported sex or CAI partners in the last 12 months. There are indications that sexual activity is an important factor contributing not only to perceived PrEP need, but also to self-selection for survey participation, suggesting that higher sexual activity is associated with a higher probability for survey participation. One aspect of this self-selection is the probability of seeing a survey recruitment message on a dating app. One of the apps we used for recruitment broadcasted each survey advertisement only for a period of 24 h. That means that frequent app users would have had a higher likelihood to see this message than infrequent users. This needs to be considered when extrapolating absolute numbers of PrEP users, intentional PrEP users, and men “in need of PrEP” based on their sexual risks, to the total MSM population of a country from proportions found in online surveys. In addition, other observations on the EMIS data suggest self-selection biases with increased sexual activity: e.g., the comparison of self-reported syphilis diagnoses in the last 12 months with syphilis cases among MSM reported to the German infectious disease surveillance system suggests a threefold higher probability that men diagnosed with syphilis had participated in the survey compared to a random distribution [personal communication by Ulrich Marcus and Susi Schink]. In the absence of an established method to estimate survey participation biases, the estimates based on sexual activity we chose are arbitrary, yet plausible. For this reason, we reported ranges with a minimum and a maximum estimated number.

A sizeable fraction of MSM indicated an intention to use PrEP if PrEP were available and affordable in a country, but did not report sexual risks defined as two or more non-steady CAI partners in the previous 12 months. These may be men who have been able to effectively manage risks, e.g., by using condoms, but who would consider switching to PrEP use as an alternative or additional risk management strategy if PrEP became available to them. Due to this group of men who would consider using PrEP, but who have also been able to manage their HIV risks effectively by using condoms, it is inherently difficult to estimate PrEP needs. We can show that this group of men with PrEP use intention and low risk reported in the past is not very different in terms of sexual partner numbers and HIV test uptake from the large group of men with no PrEP use intention and low reported risk (see Fig. S1). The groups with intention to use PrEP differ from the groups not intending to use PrEP by a slightly higher awareness of PrEP (see Table S4). How many of the men will use PrEP will likely depend on circumstances such as how convenient access to and monitoring of PrEP is, how PrEP use is advertised, how PrEP use is perceived by the gay community, how people assess risks and adverse effects of PrEP use for themselves, how satisfied these men are with the quantity and quality of the sex they have, and their expectations as to how this would change if they take PrEP. In addition, the size of this group may increase as PrEP knowledge and awareness is increasing in the MSM population.

### PrEP need estimates

With our approach to estimate PrEP needs in Germany, we arrive at estimates of approximately 52,000 (variant 2) to 66,000 (variant 1) MSM currently in need of PrEP, of which approximately 16,000 (scenario 1) to 22,000 (scenario 2) were taking PrEP as of June 2020. The estimated number of PrEP need is close to the actual estimated number of MSM diagnosed with HIV in Germany (an der Heiden et al. 2019) and would be equivalent to 7.5% of the estimated total MSM population aged 18–65 years old, or 15% of the openly gay population aged 18–65 years old.

The proportion of MSM with intention to use PrEP in 2017 that is using PrEP in 2020 may vary between approximately 63% and 88% in Berlin (or even up to 104% if we use the regional distribution of PlanetRomeo PrEP profiles, which would mean that a larger proportion of people took PrEP in Berlin in June 2020 than people indicating PrEP use intention in 2017) and is below or around 20% in the federal states with the lowest coverage. This suggests marked regional differences and persistent barriers to access PrEP for people who intend to use PrEP.

One possible barrier after costs were covered by health insurance would be limited access to health-care providers licensed to prescribe PrEP covered by health insurance, either due to geographic distance, limited number of prescribers, or inconvenience associated with scheduling an appointment, or due to individual barriers such as fear to discuss safer sex or sex in general with a health-care provider, or lack of perceived social support for taking PrEP (Mayer et al. 2020). The strong correlation between satisfied PrEP needs and number of HIV specialists per 10,000 gay men practicing in the respective federal state suggests that easy access to PrEP prescribers has indeed a large impact on meeting the needs of potential PrEP users.

For MSM in Germany, taking up PrEP requires outing oneself towards a health-care provider, just as for other medical interventions such as HIV testing. Thus, factors identified as barriers to uptake of regular HIV testing among MSM in Germany such as problems identifying and making appointments with a gay-friendly health-care service, not feeling comfortable with being out about their own sexual orientation towards friends, family members, and co-workers, or not feeling at risk of HIV infection due to perceived familiarity with sex partners will likely play a similar role for PrEP uptake (Marcus et al. 2016). In particular, the problem of identifying and making appointments with a gay-friendly health-care service is likely to play a major role, since the number of services providing PrEP prescriptions is much smaller than the number of services providing HIV testing. This assumption is supported by the strong correlation between satisfied PrEP needs and number of HIV specialists at federal state level. This correlation is exacerbated by the practical difficulties of getting PrEP prescriptions that are experienced by men not living in or near the largest cities where most of the HIV specialists practice. While the skewed geographical distribution of HIV specialists appears to have no major impact on access to treatment for HIV, this skewed distribution still appears to be a major barrier for accessing drugs for prophylaxis. This assumption regarding PrEP uptake in Germany is supported by a similar analysis of the French EMIS-2017 dataset which concludes: “(PrEP-)Eligible MSM who are not using PrEP are mostly younger, […] living in small cities, […] and more distant from preventive health care and information than PrEP users. Despite free PrEP availability in France, results suggest that PrEP is not fully accessible and that there is a need to increase PrEP demand and decentralize PrEP delivery” (Annequin et al. 2020).

If we consider MSM who might benefit from but are currently not taking PrEP, a proportion of almost 50% of them may not have had an intention to use PrEP when asked in 2017. For informed decision-making, these men may need balanced and targeted information on PrEP. They may benefit from being offered PrEP proactively and from opportunities to discuss advantages and disadvantages of taking PrEP. This would require a proactive approach including sexual history taking by health-care providers and HIV test counsellors to actively provide this information, and might encourage demand by MSM who might probably benefit from PrEP.

#### Limitations

There are several limitations to our data sources and consequently to our analysis: the self-selection bias of men intending to use PrEP if available and affordable to participate in online surveys is unknown, and our attempts to estimate this bias may be biased by halo effects. When estimating met and unmet needs for PrEP, we combine data collected by the end of 2017 with data collected in 2020. The population indicating intention to use PrEP may have changed during this time due to increased awareness of and empirical experience with PrEP use. Grouping survey participants based on reported sexual risks such as CAI is subject to recall and social desirability biases.

The extent and number of individuals with on-demand/intermittent PrEP use is difficult to estimate based on our data. The PrApp-Surveys asked for the number of pills taken per month in categories (e.g., 1–11 pills/month) and in months when PrEP was used, not collecting information on the frequency and duration of PrEP use episodes. As these parameters remain unknown, the number of intermittent or on-demand PrEP users that can be supported by a full monthly PrEP dose remains uncertain. To address this uncertainty, we calculated the impact of different assumptions in our sensitivity analyses.

## Conclusions

We believe the actual number of PrEP users in Germany by mid-2020 lies between the estimates of the two scenarios, i.e., between 15,600 and 21,600 users. The regional distribution might best be reflected by the PlanetRomeo PrEP user profile distribution. PrEP use in Germany has increased considerably between October 2017 and June 2020, but large regional inequalities persist. Outside of metropolitan areas, the proportion of people with unsatisfied PrEP needs is high and correlates with the lower density of physicians able to prescribe health insurance-covered PrEP. Thus, access to PrEP in rural areas and small to medium-sized cities needs to be addressed in the future. In the largest cities with already relatively high PrEP uptake, encouraging demand for PrEP among men with high sexual risks and no intention to use PrEP is necessary to maximize the preventive effects of PrEP.

## Supplementary Information

ESM 1 (PDF 374 kb)

## Data Availability

Most data generated or analysed during this study are included in this published article and its supplementary files. The remaining data that support the findings of this study are available from the London School of Hygiene and Tropical Medicine, but restrictions apply to the availability of these data, which were used under license for the current study and are thus not publicly available. Data are however available from the authors upon reasonable request and with permission of the London School of Hygiene and Tropical Medicine. For data requests, please contact the first author at MarcusU@rki.de.

## Footnotes

1 (a): HIV/STI checkpoints 2019–2020 (*N* = 30,097): PrEP on demand (*n* = 835) 96% MSM, 4% non-binary, Trans*; Daily PrEP (*n* = 2958) 97% MSM, 2.3% non-binary, Trans*. (b): PrEP Evaluation Project 2019-2020 (EvE-PrEP, *N* = 4620): PrEP on demand (*n* = 874) 98% MSM; Daily PrEP (*n* = 3737) 99% MSM

2 Online survey among people living with HIV in Germany in 2020 (https://www.idz-jena.de/forschung/positive-stimmen-20/)

## References

[CR1] an der Heiden M, Marcus U, Kollan C, Schmidt D, Gunsenheimer-Bartmeyer B, and Bremer V (2019) 'Schätzung der Zahl der HIV-Neuinfektionen und der Gesamtzahl von Menschen mit HIV in Deutschland, Stand Ende 2018. Epidemiologisches Bull 46:483–492

[CR2] Annequin M, Villes V, Delabre RM, Alain T, Morel S, Michels D, Schmidt AJ, Velter A, Rojas Castro D (2020). Are PrEP services in France reaching all those exposed to HIV who want to take PrEP? MSM respondents who are eligible but not using PrEP (EMIS 2017). AIDS Care.

[CR3] Apotheke adhoc (2017) “PrEP: Ratiopharm wird Preisbrecher.” https://www.apotheke-adhoc.de/nachrichten/detail/pharmazie/prep-ratiopharm-wird-preisbrecher-hiv-prophylaxe/. Accessed 01/03/2022

[CR4] BMG (2019) Schnellere Termine, mehr Sprechstunden, bessere Angebote für gesetzlich Versicherte. In Terminservice- und Versorgungsgesetz (TSVG Terminservice- und Versorgungsgesetz). https://www.bundesgesundheitsministerium.de/terminservice-und-versorgungsgesetz.html. Accessed 01/03/2022

[CR5] Bundesgesetzblatt (2019) Gesetz für schnellere Termine und bessere Versorgung (Terminservice- und Versorgungsgesetz–TSVG). https://www.bgbl.de/xaver/bgbl/start.xav?startbk=Bundesanzeiger_BGBl&start=//*%5B@attr_id=%27bgbl119s0646.pdf%27%5D#__bgbl__%2F%2F*%5B%40attr_id%3D%27bgbl119s0646.pdf%27%5D__1601365510191: Bundesgesetzblatt. Accessed 01/03/2022

[CR6] DAGNÄ (2020) Accessed 30.06.2021. https://www.dagnae.de/aerzte/

[CR7] European Medicines Agency (2016) First medicine for HIV pre-exposure prophylaxis recommended for approval in the EU. https://www.ema.europa.eu/en/news/first-medicine-hiv-pre-exposure-prophylaxis-recommended-approval-eu. Accessed 01/03/2022

[CR8] Federal Law Gazette (BGBl) (2019) Medicinal Products Act in the version published on 12 December 2005 (Federal Law Gazette [BGBl.]) Part I p. 3394, last amended by Article 11 of the Act of 6 May 2019 (Federal Law Gazette I p. 646)

[CR9] Haversath J, Gärttner KM, Kliem S, Vasterling I, Strauss B, Kröger C (2017). Sexual behavior in Germany. Dtsch Arztebl Int.

[CR10] Hayes R, Schmidt AJ, Pharris A, Azad Y, Brown AE, Weatherburn P, Hickson F, Delpech V, Noori T (2019) Estimating the 'PrEP Gap: how implementation and access to PrEP differ between countries in Europe and Central Asia in 2019. Euro Surveill 24(41):1900598 10.2807/1560-7917.ES.2019.24.41.1900598 PMC6794989 31615599

[CR11] KBV-GKV Bewertungsausschuss (2019) Beschluss des Bewertungsausschusses nach § 87 Abs. 1 Satz 1 SGB V in seiner 441. Sitzung am 14. August 2019 zur Änderung des Einheitlichen Bewertungsmaßstabes (EBM), Teil A. https://www.aerzteblatt.de/archiv/209923/Beschluss-des-Bewertungsausschusses-nach-87-Abs-1-Satz-1-SGB-V-in-seiner-441-Sitzung-am-14-August-2019-zu-Empfehlungen-zur-Vereinbarung-von-Veraenderungen-der-Morbiditaetsstruktur-nach-87a-Abs-4-Satz-. Accessed 01/03/2022

[CR12] Robert Koch-Institut (2020) Bericht zur 2. virtuellen Sitzung der Community-Vertretungen zur Evaluation der Einführung der medikamentösen HIV-Präexpositionsprophylaxe als Leistung der GKV (EvE-PrEP), Accessed 30.06.2021. https://www.rki.de/DE/Content/InfAZ/H/HIVAIDS/Bericht_Sitzung-2_Community-Vertretungen_EvE-PrEP.pdf?__blob=publicationFile

[CR13] Koelsche Blister GmbH (2017) 'PrEP-Info', Accessed 30.06.2021. https://www.koelsche-blister.de/index.php/prep/allgemeines

[CR14] Koppe U, Marcus U, Albrecht S, Jansen K, Jessen H, Gunsenheimer-Bartmeyer B, Bremer V (2019). Factors associated with the informal use of HIV pre-exposure prophylaxis in Germany: a cross-sectional study. J Int AIDS Soc.

[CR15] Marcus U, Schmidt AJ, Kollan C, Hamouda O (2009). The denominator problem: estimating MSM-specific incidence of sexually transmitted infections and prevalence of HIV using population sizes of MSM derived from internet surveys. BMC Public Health.

[CR16] Marcus U, Gassowski M, Drewes J (2016). HIV risk perception and testing behaviours among men having sex with men (MSM) reporting potential transmission risks in the previous 12 months from a large online sample of MSM living in Germany. BMC Public Health.

[CR17] Mayer KH, Agwu A, Malebranche D (2020). Barriers to the wider use of pre-exposure prophylaxis in the United States: a narrative review. Adv Ther.

[CR18] Mercer CH, Prah P, Field N, Tanton C, Macdowall W, Clifton S, Hughes G, Nardone A, Wellings K, Johnson AM, Sonnenberg P (2016). The health and well-being of men who have sex with men (MSM) in Britain: evidence from the third National Survey of Sexual Attitudes and Lifestyles (Natsal-3). BMC Public Health.

[CR19] Planet Romeo (2020) Romeos in Lockdown — Survey Results. Accessed 30.06.2021. https://www.planetromeo.com/en/blog/romeos-in-lockdown-survey-results/

[CR20] Schmidt D, Kollan C, Stoll M, Stellbrink H-J, Plettenberg A, Fätkenheuer G, Bergmann F, Bogner JR, van Lunzen J, Rockstroh J, Esser S, Jensen B-EO, Horst H-A, Fritzsche C, Kühne A, an der Heiden M, Hamouda O, Bartmeyer B, ClinSurv Study G (2015). From pills to patients: an evaluation of data sources to determine the number of people living with HIV who are receiving antiretroviral therapy in Germany. BMC Public Health.

[CR21] Scholz SM, Damm O, Elkenkamp S, Marcus U, Greiner W, Schmidt AJ (2019). Population size and self-reported characteristics and sexual preferences of men-who-have-sex-with-men (MSM) in Germany based on social network data. PLoS One.

[CR22] Weatherburn P, Hickson F, Reid DS, Marcus U, Schmidt AJ (2019) European men-who-have-sex-with-men internet survey (EMIS-2017): design and methods. Sex Res Social Policy 17:543–557

