# Supplementary material for: Analysis of HIV pre-exposure prophylaxis (PrEP) needs and PrEP use in Germany among men who have sex with men
Source: Z Gesundh Wiss. 2022 Mar 4:1–17. Online ahead of print. doi: 10.1007/s10389-022-01699-y (PMC8895693; doi:10.1007/s10389-022-01699-y)
Supplement: Supplementary file 1 — (PDF 374 kb) [file 10389_2022_1699_MOESM1_ESM.pdf]

Analysis of HIV pre-exposure prophylaxis (PrEP) needs and PrEP use among men who have sex with men in Germany

Supplemental tables and figures

Reporting of PrEP instead of TasP by MSM diagnosed with HIV

(source: Online survey among people living with HIV in Germany in 2020* (https://www.idz-jena.de/forschung/positive-stimmen-20/))

* MSM sample with non-steady partners in the last 12 months N=609
Communicating PrEP use to potential sexual partners: 70 (11.5%)
Proportion of MSM diagnosed with HIV not using online dating: 21%

Table S1a: Calculation of potential size of PrEP reporting bias in MSM online profiles in Germany.

Estimated total size of people living with diagnosed HIV in Germany	80,000
Age below 60 years	50,000
MSM	54,000
MSM below 60 years (MSM > 60 severely underrepresented in online profiles)	35,000
MSM below 60 years using online dating	30,000
Number of MSM diagnosed with HIV possibly indicating PrEP use in their online profiles	3,450

Table S1b: Calculation of number of PrEP users based on PlanetRomeo PrEP user profiles.

PrEP user profiles on PlanetRomeo as of June 2020	15,633
Subtract estimated number of profiles of MSM diagnosed with HIV	-3,450
Assumed 30% of PrEP users do not report PrEP use in their online profiles (see Table S3)	X = (15,633-3,450)*10/7
Estimated number of PrEP users based on PlanetRomeo profiles	17,400

Tab.S2: Number of PrEP pills used by PrApp survey respondents taking PrEP.

PrApp survey round	Average use per month*	daily use	intermittent use	on demand use	Total
**Round 1**	26+ days	1,212	57	12	
		97.19	23.08	4.82	
	12-25 days	31	99	52	
		2.49	40.08	20.88	
	1-11 days	4	91	185	
		0.32	36.84	74.3	
	Total	1,247	247	249	1,743
		100	100	100	
		71.54	14.17	14.29	
**Round 2**	26+ days	1,863	53	12	
		96.48	13.38	2.71	
	12-25 days	57	127	93	
		2.95	32.07	20.99	
	1-11days	11	216	338	
		0.57	54.55	76.3	
	Total	1,931	396	443	2,770
		100	100	100	
		69.71	14.30	15.99	
	**Average use per week****	**daily use**	**intermittent use**	**on demand use**	
**Round 3**	4+ days	641	38	45	
		99.69	66.67	44.55	
	0-3days	2	19	56	
		0.31	33.33	55.45	
	Total	643	57	101	801
		100	100	100	
		80.27	7.12	12.61	

* On average, on how many days per month do you take your PrEP pills?
If you don't take PrEP continuously, please indicate your average PrEP use during a month when you take PrEP

** On average, on how many days per week do you take your PrEP pills?
If you don't take PrEP continuously, please indicate your average PrEP use during a week when you take PrEP

Tab.S3: Proportion of PrApp respondents announcing PrEP use in online profile.

Type of PrEP use	PlanetRomeo			Grindr		
	PrApp round1 (n=377)	PrApp round2 (n=478)	PrApp round3 (n=151)	PrApp round1 (n=954)	PrApp round2 (n=1,605	PrApp round3 (n=391)
daily	46.0	62.1	72.0	55.1	65.0	67.5
intermittent	31.8	40.0	83.3	41.5	48.9	54.8
on-demand	17.5	45.9	33.3	32.4	46.6	65.1
Total	38.7	55.9	66.9	50.0	59.8	66.2

Table S4: Proportional regional distribution (by federal state) of all EMIS-2017 participants, EMIS-participants using PrEP, PrApp-Survey participants using PrEP, statutory health insurance PrEP prescriptions, PlanetRomeo profiles in Germany, and PlanetRomeo profiles indicating PrEP use.

	EMIS-2017 respondents with no HIV diagnosis N	Proportion using PrEP by time of data collection (11/2017) %	GayRomeo profiles by 06/2020 N	Proportion of GayRomeo profiles indicating PrEP use by 06/2020 %	PrEP intention distribution %	Distribution of PrEP-users in PrApp survey wave 2 (05/2019) (n=2,252) %	Distribution of PrEP-users in PrApp survey wave 3 (03/2020) (n=790) %	Distribution of statutory health insurance PrEP prescriptions, last quarter 2019 %	Distribution of statutory health insurance PrEP prescriptions, first two quarters 2020 %	Distribution of GayRomeo PrEP profiles by 06/2020 %	Estimate for gay population* N
Baden-Wurttemberg	2,240	1.38	69,682	1.42	11.49	7.10	7.34	5.60	5.05	6.35	44,651
Bavaria	2,672	2.02	91,857	2.34	13.70	13.77	15.95	12.20	12.10	13.76	54,061
Berlin	2,590	6.25	77,590	5.93	16.00	27.49	22.41	39.40	38.90	29.42	59,394
Brandenburg	420	1.19	10,707	1.45	2.19	0.93	1.27	0.00	0.05	0.99	8,395
Bremen	194	3.09	8,653	1.71	1.01	0.49	0.63	0.50	0.70	0.95	3,930
Hamburg	820	2.80	28,848	3.31	4.14	6.31	5.44	6.90	6.90	6.10	17,713
Hesse	1,516	2.44	53,993	2.22	7.26	8.48	9.24	8.00	8.25	7.68	31,422
Mecklenburg-West.Pomerania	318	0.31	9,785	0.97	1.41	0.53	1.14	0.20	0.15	0.61	6,329
Lower Saxony	1,422	0.98	47,838	1.42	6.93	3.60	5.70	3.10	3.10	4.34	28,175
North Rhine-Westphalia	3,842	1.80	141,360	2.27	20.14	21.54	20.76	18.80	19.35	20.50	79,376
Rhineland-Palatinate	658	1.06	27,611	1.66	3.45	2.66	3.80	0.80	0.85	2.92	13,156
Saarland	222	1.35	7,645	1.83	1.22	0.84	0.63	0.80	0.80	0.90	4,520
Saxony	1,019	0.88	26,628	1.62	4.86	4.04	3.42	2.60	2.60	2.76	20,499
Saxony-Anhalt	381	0.52	11,974	1.03	1.72	0.71	0.63	0.50	0.55	0.79	7,454
Schleswig-Holstein	681	1.62	16,334	1.29	2.91	0.84	1.27	0.20	0.30	1.35	13,395
Thuringia	364	0.27	12,818	0.71	1.57	0.67	0.38	0.30	0.35	0.58	6,956
Total	19,359	2.25	643,323	2.43	100.00	100.00	100.01	99.90	100.00	100.00	399,426

*(1,5% of adult male population, EMIS distribution, non-gay-identified MSM excluded)

Table S5: Univariable and multivariable analysis of factors associated with PrEP use intentions.

	OR	95% CI		p	aOR	95% CI		p
Demographics								
**Age**								
20 and above	ref.				ref.			
age below 20	**1.43**	1.25	1.64	0.000	**1.42**	1.21	1.67	0.000
**Settlement size**					not included			
A very big city or town (a million or more)	0.98	0.89	1.07	0.659				
A big city or town (500,000-999,999)	0.99	0.89	1.10	0.867				
A medium-sized city or town (100,000-499,999)	0.98	0.89	1.08	0.701				
A small city or town (10,000-99,999)	0.98	0.89	1.08	0.675				
Village or countryside (below 10,000)	ref.							
**Federal state**					not included			
Baden-Wurttemberg	0.91	0.81	1.03	0.148				
Bavaria	0.97	0.87	1.09	0.658				
Berlin	ref.							
Brandenburg	1.12	0.90	1.39	0.309				
Bremen	0.91	0.66	1.25	0.543				
Hamburg	**0.81**	0.68	0.96	0.017				
Hesse	0.89	0.77	1.02	0.084				
Mecklenburg-Western Pomerania	1.00	0.78	1.28	0.993				
Lower Saxony	0.91	0.79	1.05	0.203				
North Rhine-Westphalia	0.95	0.86	1.06	0.364				
Rhineland-Palatinate	0.97	0.81	1.16	0.721				
Saarland	1.03	0.77	1.38	0.823				
Saxony	0.92	0.78	1.07	0.269				
Saxony-Anhalt	1.08	0.86	1.35	0.528				
Schleswig-Holstein	0.87	0.72	1.04	0.127				
Thuringia	0.99	0.79	1.26	0.956				
**Level of education**					not included			
Low (less than secondary)	ref.							
Mid (at least upper secondary; 2-5 years post 16)	1.08	0.89	1.31	0.462				
High (first stage of tertiary or more; 6+ years post 16)	0.88	0.72	1.06	0.182				
**Partnership status**								
I have a steady partner	ref.				ref.			
I am single	**1.44**	1.35	1.53	0.000	**1.35**	1.26	1.45	0.000
I'm not sure/ it's complicated	**1.74**	1.55	1.97	0.000	**1.56**	1.37	1.79	0.000
**Sex for money**					not included			
not sold sex or less than 3 times	ref.							
Sold sex at least 3 times last 12months	**1.94**	1.58	2.39	0.000				
**Outness**					not included			
All or almost all	**0.83**	0.75	0.92	0.000				
More than half	0.89	0.79	1.00	0.050				
Less than half	0.95	0.83	1.09	0.455				
Few	1.05	0.93	1.18	0.451				
None	ref.							
Behaviours								
**Partner number**	OR	95% CI		p	aOR	95% CI		p
0-1	ref.							
2-4	**1.38**	1.27	1.51	0.000	**1.31**	1.18	1.45	0.000
5-7	**1.59**	1.44	1.75	0.000	**1.49**	1.33	1.66	0.000
8-10	**1.86**	1.65	2.10	0.000	**1.66**	1.45	1.90	0.000
11-20	**2.03**	1.84	2.24	0.000	**1.84**	1.64	2.07	0.000
21-30	**2.66**	2.30	3.07	0.000	**2.36**	2.01	2.77	0.000
31-40	**2.79**	2.26	3.44	0.000	**2.46**	1.95	3.09	0.000
41-50	**3.58**	2.72	4.71	0.000	**3.18**	2.36	4.29	0.000
>50	**4.04**	3.37	4.83	0.000	**3.51**	2.88	4.28	0.000

**Sex is always as safe as I want**								
Strongly disagree	**1.55**	1.23	1.96	0.000	**1.62**	1.24	2.12	0.000
Disagree	**2.66**	2.31	3.06	0.000	**2.16**	1.84	2.53	0.000
Neither or not sure	**1.82**	1.58	2.10	0.000	**1.91**	1.60	2.27	0.000
Agree	**1.49**	1.40	1.59	0.000	**1.36**	1.27	1.46	0.000
Strongly agree	ref.							
**Multipartner sex last non-steady partner sex**					not included			
It was me and one non-steady partner	ref.							
It was me, my partner and a non-steady partner	**0.82**	0.72	0.95	0.006				
It was me and 2 non-steady partners (a threesome)	**1.40**	1.23	1.60	0.000				
It was me and three or more other people	**1.63**	1.41	1.89	0.000				
Morbidities								
**Sexual unhappiness**					not included			
1	ref.							
2	**0.81**	0.68	0.97	0.020				
3	1.02	0.88	1.18	0.835				
4	0.99	0.85	1.15	0.868				
5	1.10	0.94	1.29	0.241				
6	1.00	0.86	1.17	0.983				
7	1.01	0.86	1.20	0.872				
8	0.97	0.82	1.15	0.708				
9	0.87	0.70	1.07	0.184				
10	1.00	0.81	1.23	0.975				
**Depression/Anxiety**								
None	ref.							
Mild	**1.16**	1.09	1.24	0.000	1.07	0.99	1.15	0.087
Moderate	**1.17**	1.05	1.31	0.005	1.02	0.90	1.17	0.726
Severe	**1.27**	1.11	1.45	0.000	**1.22**	1.04	1.44	0.015
**Sex under the influence of alcohol/drugs**								
less than half	ref.							
more than half	**1.71**	1.55	1.90	0.000	**1.35**	1.21	1.50	0.000
Interventions								
**PrEP awareness** (ever heard of PrEP)								
already heard of PrEP	ref.							
unaware	**0.81**	0.76	0.86	0.000	**0.93**	0.87	1.00	0.047
cons.					0.22	0.20	0.24	0.000

We identified the factors associated with PrEP use intention in EMIS-2017 by constructing a multivariable regression model. The univariate analysis shows that settlement size, federal state, education and sexual unhappiness have no impact on the intention to use PrEP. The multivariable regression identifies partner numbers, partnership status, perceived safe sex self-efficacy, and PrEP awareness as key factors determining PrEP use intention. Likewise, high levels of sexualised substance use, severe depression, multipartner sex, and age below 20 increase the intention to use PrEP.

Table S6: PrEP-need groups in EMIS-2017.

	intention, no reported risk*	intention and risk	no intention, but risk	no intention, no reported risk
Baden-Wurttemberg	257	232	200	1,551
Bavaria	319	264	220	1,869
Berlin	296	385	283	1,626
Brandenburg	50	43	29	298
Bremen	21	22	24	127
Hamburg	91	85	91	553
Hesse	166	143	132	1,075
Mecklenburg-Western Pomerania	30	30	32	226
Lower Saxony	163	132	113	1,014
North Rhine-Westphalia	448	409	376	2,609
Rhineland-Palatinate	72	75	50	461
Saarland	24	28	27	143
Saxony	108	99	89	723
Saxony-Anhalt	37	36	34	274
Schleswig-Holstein	61	63	73	484
Thuringia	36	31	40	257
missing	166	135	99	886
BL distributed N	2,179	2,077	1,813	13,290
Total	2,345	2,212	1,912	14,176

Fig.S1a-d: Comparison regarding CAI partner numbers, HIV test recency, disagreement with the statement "The sex I have is always as safe as I want", and PrEP awareness between the four groups 1) intention to use PrEP, low sexual risk*; 2) intention to use PrEP, moderate/high sexual risk; 3) no intention to use PrEP, moderate/high sexual risk; 4) no intention to use PrEP, low sexual risk.

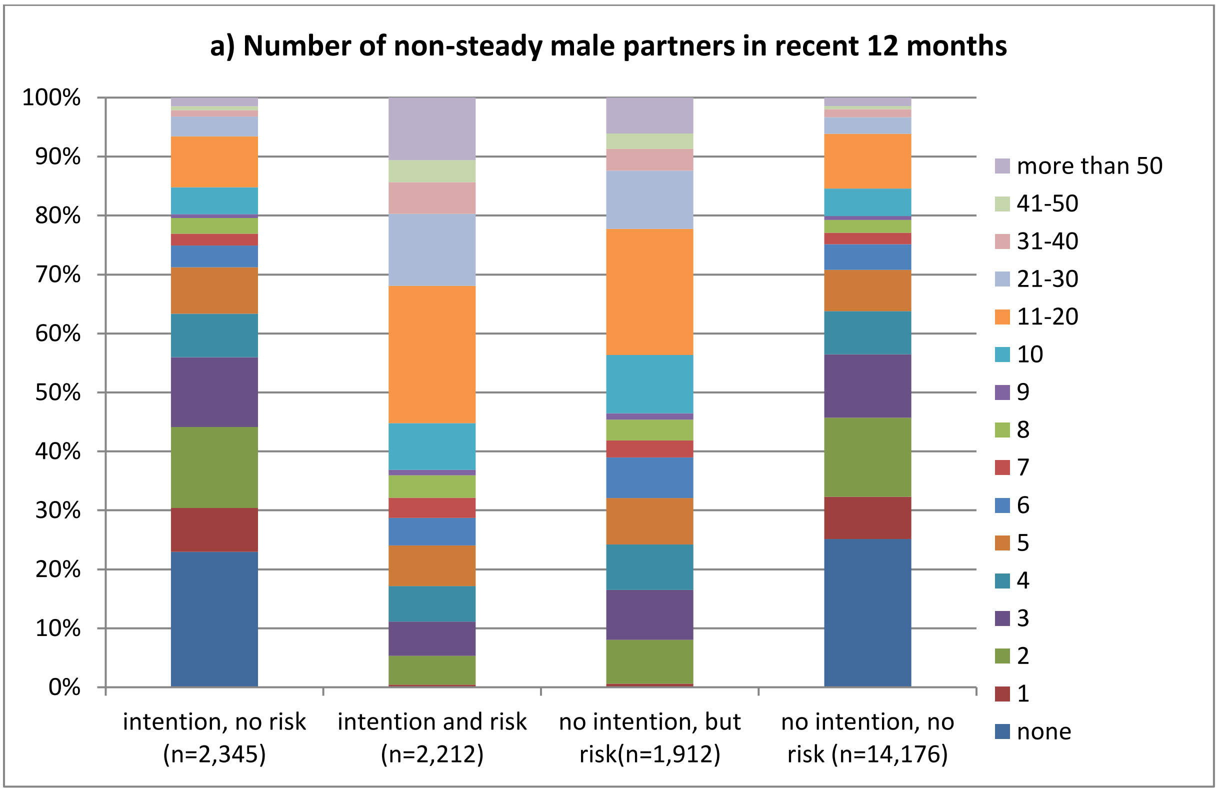

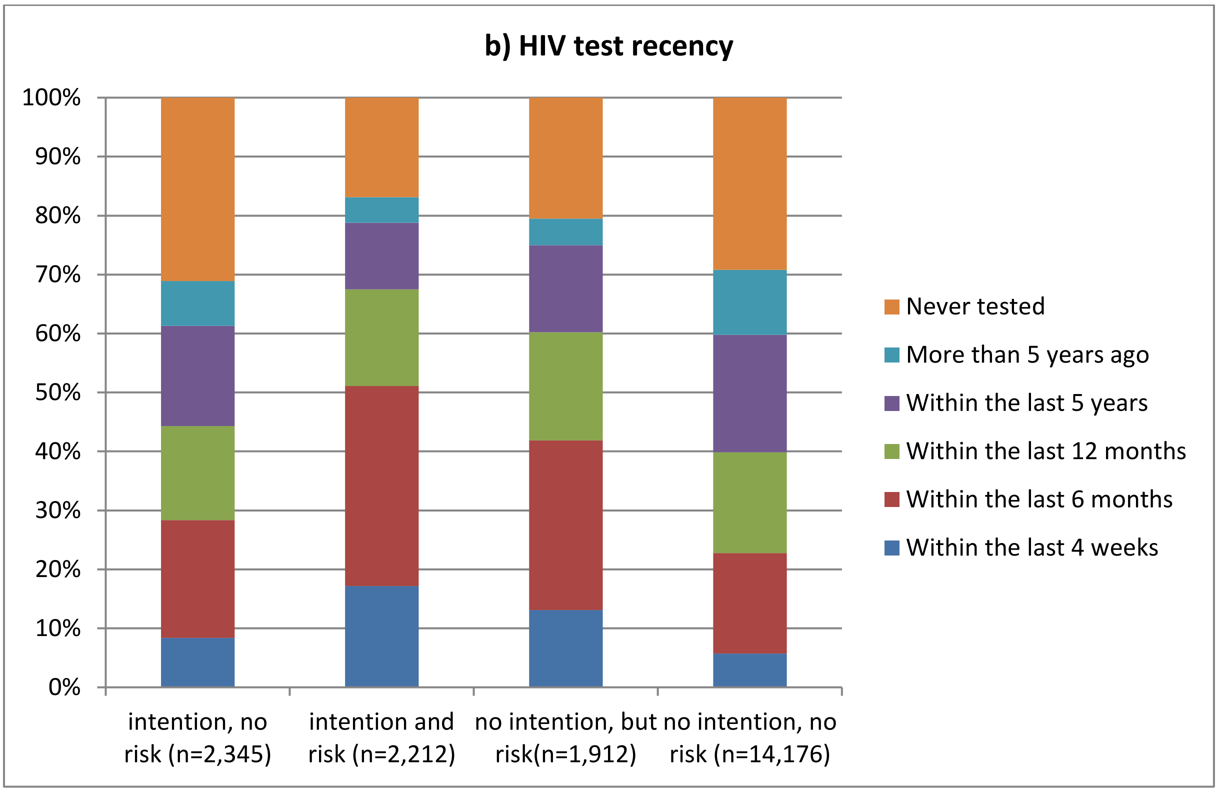
b) HIV test recency
100%
90%
80%
70%
60%
50%
40%
30%
20%
10%
0%
intention, no risk (n=2,345)
intention and risk (n=2,212)
no intention, but risk(n=1,912)
no intention, no risk (n=14,176)
Never tested
More than 5 years ago
Within the last 5 years
Within the last 12 months
Within the last 6 months
Within the last 4 weeks

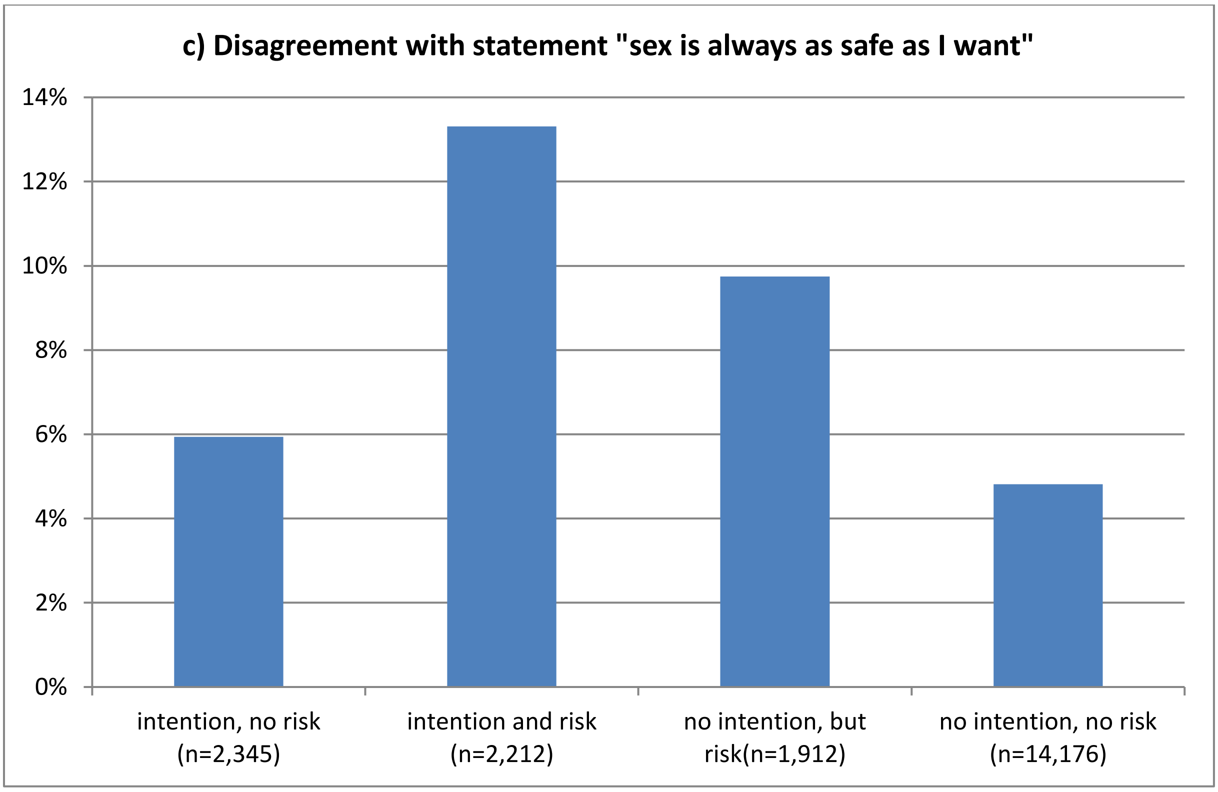
c) Disagreement with statement "sex is always as safe as I want"
14%
12%
10%
8%
6%
4%
2%
0%
intention, no risk (n=2,345)
intention and risk (n=2,212)
no intention, but risk(n=1,912)
no intention, no risk (n=14,176)

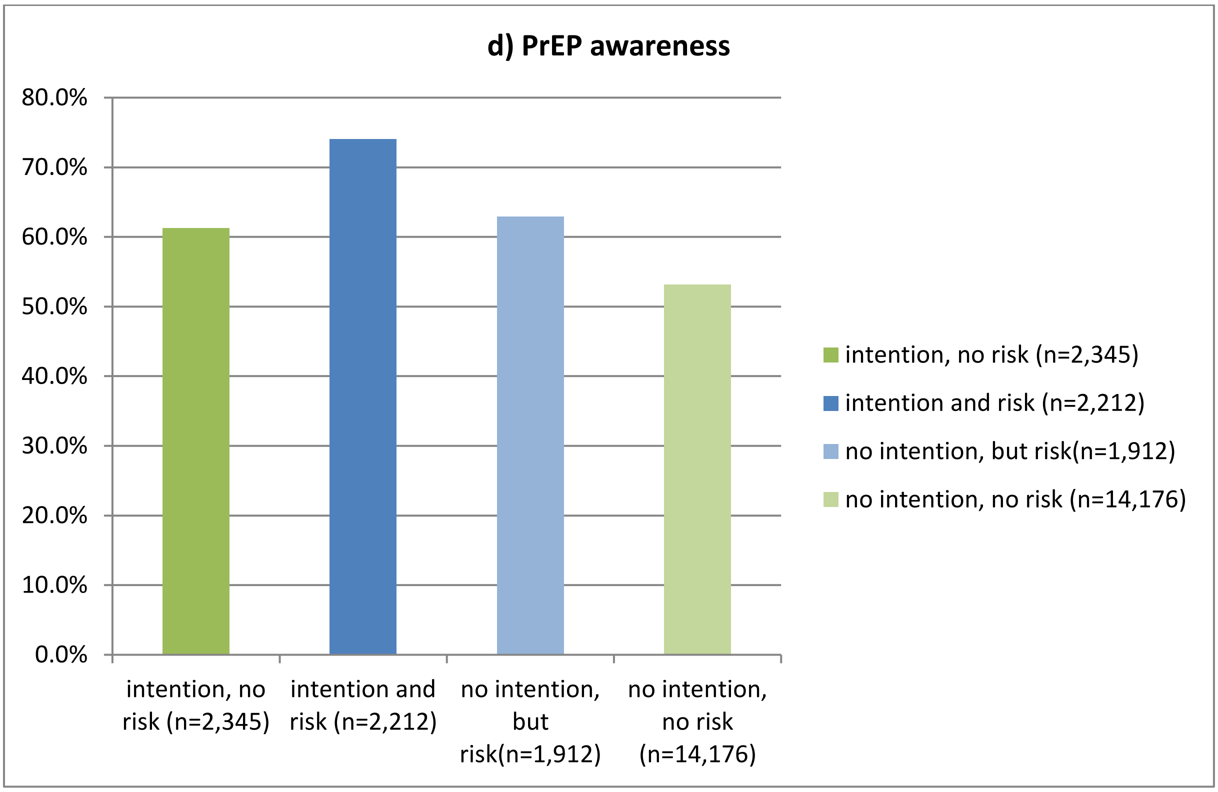

* respondents in the group „intention, no risk" who indicated less than 2 non-steady partners in the recent 12 months were *not* excluded for these figures.
